# EDUCATIONAL PROGRAM FOR THE PROMOTION OF KNOWLEDGE, ATTITUDES AND PREVENTIVE PRACTICES FOR CHILDREN IN RELATION TO TRAFFIC ACCIDENTS: EXPERIMENTAL STUDY

**DOI:** 10.1590/1984-0462/;2019;37;4;00012

**Published:** 2019-07-04

**Authors:** Carla Kalline Alves Cartaxo Freitas, Manuel Alves Rodrigues, Pedro Miguel Santos Dinis Parreira, Ana Carla Ferreira Silva dos Santos, Shirley Verônica Melo Almeida Lima, Viviane Santos Fontes, João Paulo Almeida Freitas, José Marcos de Jesus Santos, Edilene Curvelo Hora Mota

**Affiliations:** a Universidade Federal de Sergipe, Lagarto, SE, Brazil.; b Escola Superior de Enfermagem de Coimbra, Coimbra, Portugal.

**Keywords:** Child, Accidents, traffic, Health education, Accident prevention, Criança, Acidentes de trânsito, Educação em saúde, Prevenção de acidentes

## Abstract

**Objective:**

To evaluate knowledge, attitudes and preventive practices on traffic accidents in schoolchildren, before and after the implementation of a health education program.

**Methods:**

Experimental study carried out in two public schools in Northeastern Brazil. The sample was composed of 173 children from 3^rd^ to 5^th^ grade and was randomized into Experimental Group (EG; n=0) and Control Group (CG; n=8). The educational program was carried out at EG with the use of the educational therapeutic method (Health Magic Box). The data were obtained through the questionnaire Knowledge, Attitudes and Practices (KAP), applied at the beginning of the study, before any educational actions, and one month after the experimental treatment. Paired Student’s t-test was used to compare the moments before and after the intervention in the EG and initial and final evaluation in the CG.

**Results:**

The children in the EG and CG were similar in relation to sociodemographic variables, and no significant difference was observed in the level of knowledge, attitudes and preventive practices on traffic accidents between the groups in the initial evaluation. One month after the experimental treatment, a significant improvement in knowledge was observed in EG (p=0.027). Preventive attitudes and practices were also higher in children in the EG, but without significant differences in relation to CG (p=0.060 and p=0.282, respectively).

**Conclusions:**

The educational intervention increased the level of knowledge and maintained the preventive attitudes and practices on traffic accidents at the same level in 3^rd^-5^th^ grade students.

## RESUMO

**Objetivo:**

Avaliar o conhecimento, as atitudes e as práticas preventivas de acidentes de trânsito entre crianças escolares antes e depois da aplicação de um programa educativo.

**Métodos:**

Estudo experimental, com abordagens descritiva e analítica, realizado em duas escolas públicas do nordeste brasileiro. A amostra foi composta de 173 crianças do 3º ao 5º ano do ensino fundamental e aleatorizada em Grupo Experimental (GE), com 90 participantes, e Grupo Controle (GC), com 83 participantes. O programa educativo foi realizado no GE com a utilização do método eduterapêutico (Health Magic Box). Os dados foram obtidos por meio do questionário Conhecimento, Atitudes e Práticas (CAP), aplicado no início da pesquisa, antes de qualquer ação educativa, e após um mês da realização do tratamento experimental. Na análise estatística foi utilizado o teste *t* de Student pareado para comparação entre os momentos anteriores e posteriores à intervenção no GE e avaliação inicial e final no GC.

**Resultados:**

As crianças do GE e GC mostraram-se semelhantes quanto às variáveis sociodemográficas, e não foi observada diferença significativa no nível de conhecimento, atitudes e práticas preventivas de acidentes de trânsito entre os grupos na avaliação inicial. Entretanto, ainda após um mês da realização do experimento, foi evidenciada melhora significativa no conhecimento do GE (p=0,027). As atitudes e práticas preventivas também foram superiores nas crianças do GE, porém sem diferença significativa em relação ao GC (p=0,060 e p=0,282, respectivamente).

**Conclusões:**

A intervenção educativa aumentou o nível de conhecimento e manteve as atitudes e práticas preventivas de acidentes de trânsito estabilizadas em estudantes de 3º a 5º ano.

## INTRODUCTION

Traffic accidents are responsible for a large number of fatalities and injuries in several countries.^1^ According to the Global Road Safety Report, these accidents cause more than 1.3 million deaths a year and about 20‒50 million people are injured, which has a huge impact on public health and population development.^2^

The United Nations has proclaimed the period from 2011 to 2020 as the Decade of Action for Road Safety and, together with the World Health Organization (WHO), convened 178 countries to participate in a joint effort to reduce rates of traffic violence. The goal is to reduce traffic accidents worldwide by up to 50%.^3^

It is known that Europe is the region with the safest traffic in the world. In countries with stricter rules, such as the United Kingdom, Sweden, the Netherlands, Norway, and Spain, the annual deaths from traffic accidents are less than 4 per 100,000 inhabitants..^2^ The reduction of deaths in the European Union can be justified by the traffic education and awareness programs which have been in place since 1990.^4^ In this sense, it is believed that traffic education must be included in all school grades, as the behavior of today’s children and adolescents can improve the long-term statistics of traffic accidents.

Children between the ages of 5 and 10 are at the beginning of school life and are part of the vulnerability group for being run over, since sufficient pedestrian maturity, caution, and self-defense are expected in crossings.^5^ Therefore, encouraging the development of strategies in health education to prevent traffic accidents in order to guide children about the importance of appropriate pedestrian and driver behavior, should be prioritized.^6^

Thus, with the considerations presented in mind, an educational program for the prevention of traffic accidents was developed with the use of the educational terapeutic method at Universidade Federal de Sergipe (Brazil) in partnership with the Nursing School of Coimbra (Portugal). The innovation of this work is related to conducting an educational strategy for children aiming to increase their knowledge, attitudes and preventive practices on traffic accidents.

This study aimed to evaluate the knowledge, attitudes and preventive practices on traffic accidents among schoolchildren before and after the experimental treatment (educational program).

## METHOD

This is an experimental study, with descriptive and analytical approaches, carried out in the second half of 2014 in two public schools in Northeastern Brazil. The study involved the observation of dependent variables (knowledge, attitudes and prevention practices on traffic accidents) before and after an experimental treatment (educational program using the educational therapeutic method).

The eligible population was composed of 305 children, based on data provided by the management of the two schools. Thus, the sample was calculated using the formula described by Barbetta,^7^ considering a 95% confidence level and a 5% sample error, which resulted in 173 children evaluated. This sample number (n=173) was randomly distributed between Experimental Group (EG; n=90) and Control Group (CG; n=83).

The randomization of the sample was stratified according to the school and the grade of the children. Schools and classes were randomized and classified into EG, eligible for the educational program, and CG, not eligible for intervention. Each school was assigned, for the purposes of randomization, a sequence of numbers between one and eight. Using random.org and based on the simple random sampling technique, two numbers were chosen to define the schools participating in the study.

Each school had a 3^rd^, 4^th^ and 5^th^ grade class. In order for CG and EG to be equivalent in terms of the age of the participants and their grade, a number was given for each class according to the school they attended:

- 3^rd^ grade: school 1 and school 2.
- 4^th^ grade: school 1 and school 2.
- 5^th^ grade: school 1 and school 2.

Thus, it was designated that institution with number 1 would be part of the EG, and number 2 would be in the CG, resulting in three classes in the EG and three classes in the CG.

The data collection instrument was Knowledge, Attitudes and Practices (KAP), a standardized questionnaire to obtain diagnoses that precede an educational intervention, aiming to guide this action in an oriented, systematized and plausible way, once it unveils the participants’ knowledge (what they know), attitudes (what they think) and practices (what they do) on a certain subject.^8^ It consists of four parts. The first one contains the sociodemographic data, and the other three are related to knowledge (14 questions), attitudes (12 questions) and practices (six questions). The evaluation was performed by the proportion of correct answers. For each correct answer, a score of 1 was assigned, and thus the sum of each part of the questionnaire was obtained.

All questions in the KAP were adapted to approach the theme of traffic accident prevention, based on the most recent literature on the subject, one the validation of the instrument by three experts in the area, and a pilot test conducted with 15 children who did not participate in this research. The instrument contemplates items of the educational program and also addresses the risk and protection factors of the need for preventive behaviors, solidarity and empathy from the child and their justification.

Data collection took place in three stages. In the first one, the pre-test was carried out, with application of the KAP questionnaire with the EG and the CG. The next day, in the second phase, the educational intervention was done with all students in the EG. The third stage occurred only one month after the educational activity took place, and consisted of a new application of the KAP questionnaire to all the study participants; in addition, each student was given a folder with the summary of the information offered, in order to reinforce the knowledge addressed in the activity.

The data were tabulated in Microsoft Office Excel (2010) and then imported into the Statistical Package for Social Sciences 20.0 for Mac (SPSS Inc., Chicago, Illinois, United States) for descriptive and analytical statistical analyzes. Numerical variables were expressed as mean and standard deviation, and categorical variables were described by means of absolute and relative frequencies. Fisher’s exact test was used to investigate associations between categorical variables with low-frequency cells. In the comparison of the quantitative variables between the EG and the CG, Student’s t-test was used for independent variables. The paired Student’s t-test was used to compare the moments before and after the intervention in the EG and initial and final evaluation in the CG. In all cases, a significance level of <0.05 was used.

The research was approved by the Research Ethics Committee of Universidade Federal de Sergipe with the following Certificate of Presentation for Ethical Assessment (CAAE): 16003813.9.0000.5546 (protocol nº 298.534). The identity and rights of the participants of this study were preserved, in compliance with Resolution 466/2012 of the National Health Council, in Brasilia, DF, Brazil.^9^ Those responsible for the children signed the informed consent with a guarantee of refusal at any time, without prejudice from the institutions.

The educational intervention was carried out in the EG by means of the educational method, and its planning and organization were carried out in Portugal, with the participation of the method’s creator.^10^ It includes several techniques and has already been studied and implemented in several actions with schoolchildren, proving to be effective. For this investigation, the method was adapted to prevent traffic accidents and the Health Magic Box (HMB) technique, developed by Rodrigues, was used.^10^

The educational therapy seeks to stimulate the child’s willingness to point out doubts or to comment on ideas about specific issues related to their own perception of traffic (questioning) from the multiple suggestions in the image.^10^ ^,^ ^11^ Children’s drawings are facilitators of the interaction between educators, health professionals and children, as well as being effective technical and educational tools in planning and action.^12^

HMB is an analogy to the human brain, an extraordinary library of wonderful images arranged in dispositional patterns and ready to be evoked in the future. Each HMB addresses a health issue and has a specific purpose with the use of script. The content of HMB varies according to the objective and maintains the same functional principle.^13^

An educational therapeutic script is a technical and educational construct organized around a health topic (in this case, the prevention of traffic accidents) directed at children, within a delimited time (usually 90 minutes), through educational therapeutic regulation and feedback, from which a change of attitude and behavior is expected. For this study, only one script was developed and applied, as it exclusively addressed the theme of traffic accident prevention. This script consisted of 10 cards, nine of which dealt with traffic problems and the last one dealt with the children’s commitment to change.

The script was carried out as follows:

- On one side of the box were placed small cards with meaningful drawings created by the children (in the first phase of the research) regarding their perception on the prevention of traffic accidents. On the other side, a question was carefully selected (ten cards can be placed in the box, with the last one being aimed at a voluntary change of attitude or health behavior - in this case, behavior to prevent traffic accidents).
- The drawings and questions were chosen and validated by some scientific criteria:^10^ ^,^ ^13^ ^,^ ^14^

*Purpose:* messages organized in meaningful patterns give multiple suggestions, which is intentionally directed towards the promotion of positive behaviors in relation to traffic;

*Meaning:* the drawings were made by the children, the ideas were theirs, and so, are significant to them. In the process of visualizing the previously selected images, the child was stimulated to interact with expressive messages that are familiar to them and with ideas that are meaningful to them, generating a mirror effect;

*Adequacy:* each image was anchored on issues that are appropriate to the child’s cognitive developmental stage;

*Hierarchy:* the issues were discussed in a certain sequence to facilitate understanding;

*Scientific evidence:* the content of the questions was based on scientific results, and the content of the script, based on materials made available by the following traffic agencies: Community and Transit: Educating for Traffic and National Road Safety Observatory, in addition to the results of drawings made by the children themselves.

- The cards were drawn from the box by the children, in logical order, and they searched in groups for answers to each question.
- The questions were read and discussed by the children, with mediation from the health professional and the teacher.
- At the end of the educational therapeutic feedback, the children withdrew the last card, which indicated preventive behavior, and were invited to practice the indicated behavior and to present the results 30 days after the intervention.

## RESULTS

Children from the EG and from the CG were similar regarding the sociodemographic variables. The mean age of the EG participants was 9.6±1.2 years and the mean age was 9.8±1.2 years. More than half were male (EG: 51.7% and CG: 53.4%) and studied in the morning shift (EG: 68.9% and CG: 100%). In relation to the grade, the following proportional distribution was observed:

- 3^rd^ grade: EG: 36.7% and CG: 31.3%.
- 4^th^ grade: EG: 32.2% and CG: 36.1%.
- 5^th^ grade: EG: 31.1% and CG: 32.5% (Table 1).

When questioned about the existence of some form of transportation in their household, 86.5% (n=77) of the children in the EG and 86.6% (n=71) of the CG responded positively to the question. Most of them used bicycles at the time of the research (EG: 83.3% and CG: 84.1%), and many performed their daily commute to school unaccompanied by an adult and on foot (EG: 35.2 % and CG: 29.3%) (Table 1).

**Table 1:** General characterization, comparison of sociodemographic characteristics and form of transportation of schoolchildren according to the experimental group and the control group. Sergipe, Brazil, 2014*.

	Group		p-value
	EG (n=90)	CG (n=83)	
Age*	9.6±1.2	9.8±1.2	0.290
Sex			
Male	46 (51.7)	43 (53.4)	0.921
Female	43 (48.3)	39 (47.6)	
School grade			
3^rd^	33 (36.7)	26 (31.3)	0.747
4^th^	29 (32.2)	30 (36.1)	
5^th^	28 (31.1)	27 (32.5)	
Time of classes			
Morning	62 (68.9)	83 (100)	<0.001
Afternoon	28 (31.1)	0 (0)	
Existence of some form of transportation in the household			
Yes	77 (86.5)	71 (86.6)	0.990
Form of transportation present in the household			
Car	6 (7.6)	15 (20)	0.248
Motorcycle	22 (27.8)	13 (17.3)	
Bicycle	8 (10.1)	11 (14.7)	
Car and motorcycle	4 (5.1)	2 (2.7)	
Car and bicycle	8 (10.1)	8 (10.7)	
Motorcycle and bicycle	15 (19)	11 (14.7)	
Car, motorcycle and bicycle	16 (20.3)	15 (20)	
Rides a bicycle			
Yes	75 (83.3)	69 (84.1)	0.885
Form of commuting to school			
On foot	31 (35.2)	24 (29.3)	0.759
On foot accompanied by an adult	18 (20.5)	20 (24.4)	
Using some form of transportation	38 (43.2)	36 (43.9)	
Other	1 (1.1)	2 (2.4)	
Has ever participates in an educational activity on traffic accidents?			
Yes	86 (95.6)	73 (89)	0.106
No	4 (4.4)	9 (11)	

*Unanswered/ignored items were suppressed from the analysis; EG: experimental group; CG control group. Note: values expressed as mean and standard deviation.

Almost all of the children reported having participated in some educational activity on traffic accident prevention before the intervention in the present study (EG: 95.6% and CG: 89%). Possibly, because of this, there was no difference in the level of knowledge, attitudes and preventive practices between the EG and the CG in the initial evaluation. However, even after one month of the experimental treatment (educational program), a significant improvement in the knowledge of the EG was evidenced (p=0.027). Preventive attitudes and practices were also higher in children in the EG; however, without significant difference in relation to CG (p=0.060 and p=0.282, respectively) (Table 2).

**Table 2:** Comparison of knowledge, attitudes and practices in initial (pre) and final (post) assessments among groups of schoolchildren. Sergipe, Brazil, 2014.

	EG n=90	CG n=83	p-value
Knowledge-Correct-Pre	9.7±2.3	10.3±2.2	0.110
Attitudes-Adequate-Pre	9.2±2.3	9.4±2.4	0.559
Practices-Adequate-Pre	4.1±1.1	4.3±1.1	0.272
Knowledge-Correct-Post	10.7±2.5	9.8±3.1	0.027
Attitudes-Adequate-Post	9.5±2.7	8.6±3.1	0.060
Practices-Adequate-Post	4.1±1.2	3.9±1.2	0.282

EG: experimental group; CG: control group. Note: values expressed as mean and standard deviation.

Table 3 presents a significant difference (p=0.003) in the EG regarding the students’ level of knowledge. There was no significant difference between groups regarding attitudes and practices (p=0.320 and p=0.948, respectively).

**Table 3:** Comparison before and after the intervention regarding the correct knowledge, and adequate attitudes and practices regarding the prevention of traffic accidents among the participants of the experimental group. Sergipe, Brazil, 2014.

Experimental group (n=90)	Initial assessment	Final assessment	p-value
Knowledge-Correct	9.7 ±2.3	10.7±2.5	0.003
Attitudes-Adequate	9.2±2.3	9.5±2.7	0.320
Practices-Adequate	4.1±1.1	4.1±1.2	0.948

Note: values expressed as mean and standard deviation.

The data in Table 4 show that in the CG, regarding the children’s knowledge, the number of correct answers in the initial evaluation (10.3±2.2) was higher than in the final evaluation (9.8±3.1), but without significant difference (p=0.085). The final evaluation also showed a reduction in the level of appropriate attitudes and practices with significant differences, respectively, p=0.033 and p=0.003.

**Table 4:** Comparison of the initial and final assessment of the correct knowledge, and appropriate attitudes and practices regarding the prevention of traffic accidents among participants in the control group. Sergipe, Brazil, 2014.

Experimental group (n=83)	Initial assessment	Final assessment	p-value
Knowledge-Correct	10.3 ±2.2	9.8±3.1	0.085
Attitudes-Adequate	9.4±2.4	8.6±3.1	0.033
Practices-Adequate	4.3±1.1	3.9±1.2	0.003

*Values expressed as mean and standard deviation.

## DISCUSSION

According to Article 76 of the New Brazilian Traffic Code, traffic education must be promoted in pre-school and in primary and secondary education through coordinated actions between agencies and entities of the National Traffic and Education System Union, the Federal District, the states and municipalities, in their respective fields of action.^15^

Currently in the Americas, many families still use motorcycles as a form of land transportation,^16^ and, in recent analysis, mortality rates related to the use of these vehicles have increased significantly in all subregions of the continent.^17^ In addition, although it is known that adults are the drivers of these vehicles, working on preventive actions with children would eventually allow them to be multipliers for their families, since the lack of knowledge from parents and legal guardians on road traffic safety with children.^18^

A high ratio of use of bicycles was identified among the children in the present study, which stresses the importance of encouraging and work their safe use so that they become conscious adults and decrease the index of cars used by the population in general. In several developed countries, there is a strong incentive to use the bicycles with many justifications, including reducing pollution and reducing traffic accidents.^16^ ^,^ ^19^

In Denmark, Germany and the Netherlands, bicycle use is widely encouraged due to the drastic reduction of traffic-related accidents and fatalities. These countries establish various rights for cyclists, have adequate parking facilities, full integration with public transportation, education for comprehensive traffic and training of cyclists and drivers, as well as promotional events designed to generate public enthusiasm towards supporting cycling.^20^

It was observed that the majority of the children commute to and from school unaccompanied by an adult, signaling a possible lack of concert or preparation from the parents in this sense. It is essential to guide these children regarding preventive behavior during the commute, since the proximity between the school and the residence does not reduce the risk of possible accidents in this public.^21^

Many children answered that they participated in an educational activity about traffic accident prevention at school prior to the intervention performed in the present study. Relevant to this fact, the managers of these institutions confirmed that, in the academic calendar, the month of September is dedicated to the National Traffic Week program, a period when most Brazilian schools approach the theme in the classroom.^22^ The questionnaire was applied two months after this event, which may explain why most of the students pointed this out.

Expanding children’s awareness of traffic is another recommendation for stepping up traffic safety.^23^ By promoting the intervention of the present study, reinforcing the prevention theme, there was an increase in the knowledge of the children of the EG and establishment of appropriate preventive attitudes and practices. The objectives of traffic education are achieved in the long term. Thus, to promote change in attitudes and practices, it is necessary that the intervention be performed on a regular basis.^24^

It is assumed that because National Traffic Week happened shortly before the initial evaluation of this study, children could be sensitized, and the correct knowledge, the appropriate attitudes and practices of the children in the CG were at a level equivalent to those in the EG. However, the lack of educational intervention developed by this research two months after the event caused a reduction in the level of all variables analyzed in the CG. This reveals the importance of the implementation of regular programs around the theme of traffic in schools.

Finally, it could be inferred that educational intervention with the use of the educational therapeutic method positively influenced the knowledge, attitudes and practices of the children in the EG on the theme of prevention of traffic accidents. A limitation of present study is the lack of follow-up of the children through the application of the KAP questionnaire in the subsequent months, in order to confirm if the educational activity would permanently influence the variables evaluated.

It should be emphasized that there was no difficulty in implementing the method in the Brazilian context, and its use is fundamental in order to promote an educational culture towards prevention of traffic accidents among children. It is believed that the method can be used in new educational contexts and allows immersion in the local culture, while creating a space for dialogue and enlightenment that facilitates the exchange of knowledge among those involved.

It is recommended that periodic training be carried out among health professionals involved in the education of children about the use of the educational method in order to promote preventive behavior in relation to traffic.
