# ERRATUM

**DOI:** 10.1590/1984-0462/;2019;37;4;00012erratum

**Published:** 2021-07-16

In the manuscript “Educational program for the promotion of knowledge, attitudes and preventive practices for children in relation to traffic accidents: Experimental study”, DOI: 10.1590/1984-0462/;2019;37;4;00012, published in the Rev Paul Pediatr. 2019;37(4):458-64, on page 463.

**Where it reads:**

Funding

This study did not receive funding.

**It should read:**

Funding

This study was ﬁnanced in part by the Coordenação de Aperfeiçoamento de Pessoal de Nível Superior - Brasil (CAPES) - Finance Code 001.

